# Interplay between CD8α^+^ Dendritic Cells and Monocytes in Response to *Listeria monocytogenes* Infection Attenuates T Cell Responses

**DOI:** 10.1371/journal.pone.0019376

**Published:** 2011-04-29

**Authors:** Dilnawaz Kapadia, Aida Sadikovic, Yannick Vanloubbeeck, Dirk Brockstedt, Lawrence Fong

**Affiliations:** 1 Division of Hematology/Oncology, Department of Medicine, University of California San Francisco, San Francisco, California, United States of America; 2 Aduro Biotech, Berkeley, California, United States of America; French National Centre for Scientific Research, France

## Abstract

During the course of a microbial infection, different antigen presenting cells (APCs) are exposed and contribute to the ensuing immune response. CD8α^+^ dendritic cells (DCs) are an important coordinator of early immune responses to the intracellular bacteria *Listeria monocytogenes* (Lm) and are crucial for CD8^+^ T cell immunity. In this study, we examine the contribution of different primary APCs to inducing immune responses against Lm. We find that CD8α^+^ DCs are the most susceptible to infection while plasmacytoid DCs are not infected. Moreover, CD8α^+^ DCs are the only DC subset capable of priming an immune response to Lm *in vitro* and are also the only APC studied that do so when transferred into β2 microglobulin deficient mice which lack endogenous cross-presentation. Upon infection, CD11b^+^ DCs primarily secrete low levels of TNFα while CD8α^+^ DCs secrete IL-12 p70. Infected monocytes secrete high levels of TNFα and IL-12p70, cytokines associated with activated inflammatory macrophages. Furthermore, co-culture of infected CD8α^+^ DCs and CD11b+ DCs with monocytes enhances production of IL-12 p70 and TNFα. However, the presence of monocytes in DC/T cell co-cultures attenuates T cell priming against Lm-derived antigens *in vitro* and *in vivo*. This suppressive activity of spleen-derived monocytes is mediated in part by both TNFα and inducible nitric oxide synthase (iNOS). Thus these monocytes enhance IL-12 production to Lm infection, but concurrently abrogate DC-mediated T cell priming.

## Introduction

Host defenses against intracellular pathogens, including the gram-positive bacterium *Listeria monocytogenes* (Lm), require coordinated interactions between a number of innate and adaptive components to clear an infection (reviewed in [1], [2], [3], [4]). The mouse model for Lm infection shows that protective immunity requires a complex interplay between a number of innate effectors including neutrophils, macrophages and NK cells [5], [6], [7], [8], [9], [10], [11]. Both Interferon gamma (IFNγ) (primarily from NK cells) and tumor necrosis factor alpha (TNFα) are essential for early resistance to infection [11], [12], [13], [14]. Innate defenses against Lm were shown to depend on **TNFα** and **i**nducible nitric oxide synthase (iNOS) **p**roducing **DCs** (TipDCs) (the precursors of which may be monocytes) [5], [6], [15]. On the other hand, secretion of Type I IFNs upon cytosolic entry by Lm appears to impair the response to Lm [15], [16], [17]. These innate cells are required early for host survival and bacterial clearance [5], [6], [7], [8], [9], [10], [11], [18], while development of adaptive immunity and immunologic memory requires lymphocytes such as CD4^+^ and CD8^+^ T cells, the latter being crucial for long-term protection from subsequent exposures.

At the crossroads of innate and adaptive immunity are DCs, and in the context of host-pathogen interactions the major subsets appear to be CD8α^+^ DCs, CD11b^+^ DCs and plasmacytoid DCs (PDCs) (Reviewed in [19], [20], [21], [22]). Lm-specific adaptive responses have been demonstrated to require DCs [23], and studies have shown that DCs themselves can be early targets (within 3–6 hrs) of Lm in the spleen [24], [25]. Alternatively, Lm can be initially taken up by monocytes, macrophages, and neutrophils to trigger an innate immune response. Antigen from these infected cells may then be taken up by DCs, and subsequent priming of CD8^+^ T occurs via cross-presentation of these acquired antigens by CD8α^+^ DCs. Consistent with this hypothesis, CD8α^+^ DCs specifically have been implicated in both early bacterial clearance [25], and in priming of T cells to Lm–encoded antigens [26].

Nevertheless, while it was believed that the DC subset with the capacity to cross-prime antigens is primarily the CD8α^+^ DCs [26], [27], there are studies that suggest other DC subsets may also be capable of cross-presentation [28], [29], [30]. Furthermore, whether Lm can directly infect specific DC subsets and if these DCs can activate naïve T cells remains unresolved. Given the reports of T cell activation in the absence of CD11c^+^ cells *in vivo* [25], we hypothesized that different antigen presenting cells (APCs) could make varying contributions to induction of Lm-specific immunity. Finally, the interplay between different APCs in priming of adaptive immune responses has not been elucidated.

We demonstrate that CD8α^+^ DCs are the most susceptible to infection *ex vivo* and the only subset capable of priming antigen specific T cells to Lm. CD11b^+^ DCs, while elaborating cytokines in response to infection, did not elicit a strong CD8 T cell response, and PDCs were relatively refractory to infection *ex vivo*. Lm infected monocytes secreted high levels of TNFα and IL-12 p70, exhibiting key characteristics of inflammatory monocytes [31]. Nevertheless, we find that the presence of these monocytes can modulate the effector function and potency of CD8α^+^ DCs by enhancing DC production of cytokines, but paradoxically inhibiting T cell activation. Further analysis into the possible mechanisms involved in this inhibition of T cell priming revealed that TNFα and iNOS appear to be key players in this inhibition as blockade of iNOS or TNFα each resulted in loss of this inhibition. These findings underscore the complex coordination of both the innate and adaptive players in immunity against Lm.

## Results

### CD8α^+^ DCs are most susceptible to direct Infection by Lm

**Figure 1:**
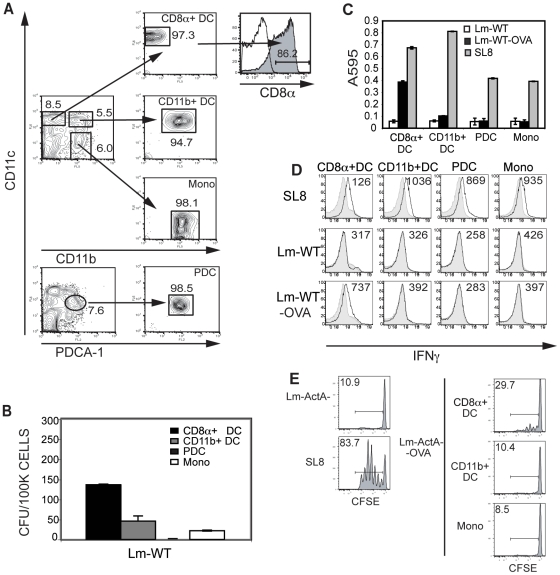
CD8α^+^ DCs are most susceptible to Lm infection and elicit antigen specific T cell responses. (**A**) Splenocytes from Flt3-L mobilized mice were stained with CD11b, PDCA-1 and CD11c; and sorted on a MoFlo sorter. CD8α^+^ DCs were sorted as CD11c^hi^ CD11b^−^ PDCA-1^−^; CD11b^+^ DCs as CD11c^hi^ CD11b^+^ PDCA-1^−^; and PDCs as CD11c^int^ CD11b^−^ PDCA-1^+^. Monocytes were sorted as CD11c^−^ CD11b^+^ PDCA-1^−^. CD8α^+^ DCs were stained with CD8α to confirm expression. (**B**) Sorted DC subsets were infected with Lm-WT or Lm-WT-OVA at an MOI of 1 for one hour and incubated in media containing gentamicin for 1 hr at 37°C. Lm-WT infected cultures were then lysed and plated on BHI agar plates overnight at 37°C. Lm colonies forming units (CFU) were enumerated. (**C**) Infected DCs were co-incubated overnight at 37°C with the LacZ inducible B3Z T cell hybridoma. (**D**) Infected DCs were incubated at 37°C with purified naïve OT-1 transgenic T cells for 4 days. On D4 T cells were washed and restimulated with B6 splenocytes pulsed with SL8 peptide for 5 hours in the presence of brefeldin A. T cell activation was assessed intracellular staining (ICS) for IFNγ in response to the denoted uninfected APCs (shaded histograms) or APCs either loaded with SL8 peptide or infected with Lm where indicated (solid line). Numbers represent geometric mean fluorescence intensities (MFI). (**E**) CD8^+^ OT1 T cells were isolated and labeled with 5 mM CFSE. 1×10^6^ cells were adoptively transferred intravenously (i.v.) into naïve B6 mice. Sorted CD8α^+^ DCs, CD11b^+^ DCs or monocytes were infected with either Lm-actA^−^, Lm-actA^−^-OVA or pulsed with 1 mM SL8 peptide and were injected subcutaneously (s.c.) into the foot-pad 8 hours later. Popliteal lymph nodes were harvested at 60 hrs, and proliferation was determined via CFSE dilution assessed by flow cytometry. Numbers represent percent divided cells. Data are representative of at least three independent experiments.

The majority of studies on DC infection with Lm rely on *in vitro*-generated, bone marrow-derived DCs (BMDCs) [32], [33], but these DCs may not reflect DCs *in vivo*. Hence, for this study, primary APCs were isolated from B6 mice following FMS like Tyrosine kinase 3 ligand (Flt3-L) mobilization with a B16 cell line transduced to secrete Flt3-L (B16/Flt3-L) [34], [35]. Flt3-L being a growth factor for DCs, using the B16-Flt3L cell line allowed us to expand DCs and monocytes to sufficient numbers *in vivo*. On D12–16 post injection, DC subsets were isolated by flow cytometry-based sorting. CD8α^+^ DCs were sorted as CD11c^hi^ CD11b^−^ PDCA-1^−^, CD11b^+^ DCs were sorted as CD11c^hi^ CD11b^hi^ PDCA-1^−^, and PDCs were sorted as CD11c^int^ CD11b^−^PDCA-1^+^. Spleen-derived monocytes were isolated as CD11b^+^ CD11c^−^. CD11c^hi^ CD11b^−^ PDCA-1^−^ DCs expressed high levels of CD8α (Figure 1A). We found that the DCs isolated from Flt3-L mobilized mice expressed levels of CD40, CD80, CD86 and MHC II comparable to DCs in unmanipulated mice (Figure S1).

These sorted primary DC subsets were infected *ex vivo* with the wild-type strain of Lm. Infections were performed with agitation in order to minimize differences between subsets due to cell adherence. Of the primary DC subsets the CD8α^+^ DCs were the most highly infected and exhibited titers higher than seen in the monocyte fraction (Figure 1B). CD11b^+^ DCs were infected at lower levels and PDCs appeared remarkably refractory to infection. Surprisingly, primary monocytes isolated were not as highly infected as the CD8α^+^ DCs, although these monocytes may be more efficient at killing intracellular bacteria, resulting in lower CFUs.

### CD8α^+^ DCs present Lm-derived antigen

We next sought to compare the ability of the DC subsets to present Lm-derived antigens and to test whether interactions with DC and monocytes have any effect on T cell activation. For these experiments, DC subsets were infected for 1 hr with Lm strains engineered to express OVA SIINFEKL (Lm-WT-OVA). Cells were washed and incubated overnight in gentamicin containing media in the presence of B3Z T cells, a LacZ-inducible T cell hybridoma that recognizes OVA_257–264_ SIINFEKL (SL8) [36]. CD8α^+^ DCs were most efficient at presenting antigen to the B3Z CTLs. No significant T cell activation was detected from the infected CD11b^+^ DC or PDC subset or from monocytes in this assay (Figure 1C). While both CD8α^+^ DC and CD11b^+^ DC subsets demonstrated comparable ability to present SL8 peptide, only the CD8α^+^ DCs were able to present Lm-derived OVA antigen.

To assess the capacity of infected DCs to prime naïve antigen-specific T cells *in vitro*, isolated APCs were again infected with Lm-WT-OVA and now co-cultured with purified naïve OT1^+^ CD8^+^ T cells, which recognize SL8 peptide in the context of H-2K^b^ [37]. IFNγ secretion from these CD8^+^ T cells was assayed via intracellular cytokine staining (ICS). In concurrence with the antigen presentation result, CD8α^+^ DCs were most potent in priming antigen-specific T cells (Figure 1D). There was no IFNγ detected from CD11b^+^ DCs or monocytes despite low levels of infection.

We used an adoptive transfer system using CFSE labeled purified OT1^+^ T cells, to study T cell priming in response to Lm infection *in vivo*, Labeled T cells were adoptively transferred into wild-type B6 mice that were subsequently primed subcutaneously with CD8α^+^DC, CD11b^+^ DC or monocytes infected with Lm *ex vivo*. In order to limit spread of the bacteria from the *ex vivo* infected DCs to other host DCs we used the Lm-ActA^−^ and Lm-ActA^−^-OVA strains for infection in these experiments since this strain is deleted in the *ActA* gene required for cell-to-cell invasion. While spread of bacteria is still possible due to dissemination from the death of infected APCs, we hoped to limit at least part of the mechanism of bacterial spread. Consistent with our *ex vivo* data, only infected CD8α^+^ DCs were able to elicit T cell proliferation in response to Lm-ActA^−^-OVA (Figure 1E). There was no detectable CFSE dilution in response to infected CD11b^+^ DCs or monocytes, supporting the notion that these subsets are not capable of directly priming T cells, nor can they effectively provide a source of antigen that could be subsequently presented by endogenous host APCs.

### Primary DCs infected *ex vivo* with Lm secrete low levels of cytokine

**Figure 2:**
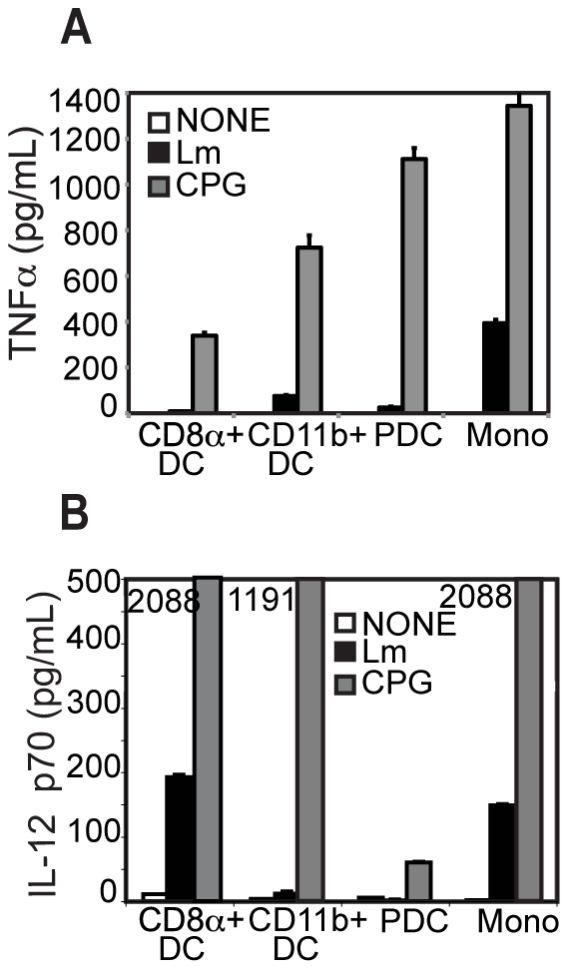
Cytokine production by APCs in the presence of IFNγ. Sorted DC subsets were infected with Lm-WT and incubated overnight at 37°C in the presence of gentamicin and 100 U/ml IFNγ. At 18 to 20 hours post infection supernatants were assayed for (**A**) TNFα and (**B**) IL-12 p70 production by ELISA. Data are representative of at least three independent experiments.

Next we assessed the capacity of different APCs to produce cytokine in response to Lm infection. DC subsets were again isolated and infected *ex vivo*. 18 hrs post infection, culture supernatants were harvested and assayed for TNFα, IL-12 p70, and IFNα. Cytokine secretion from all DC subsets and monocytes was very low (data not shown) compared to published results with *in vitro* generated DCs such as BMDCs. Given the requirement of IFNγ for bacterial clearance and the fact that NK cell involvement in the innate response could provide an initial burst of IFNγ, we cultured these primary DCs in the presence of IFNγ. Upon infection, low levels of TNFα were detected from CD11b^+^ DCs and more significant levels of IL-12 p70 were detected from CD8α^+^ DCs (Figure 2 A, B). There was also significant secretion of IL-12 p70 and TNFα from monocytes. None of the APCs studied, including PDCs, secrete detectable levels of IFNα and the addition of IFNγ had no effect on IFNα production (data not shown).

### Interaction between DCs and monocytes during Lm infection *ex vivo* enhances cytokine production

**Figure 3:**
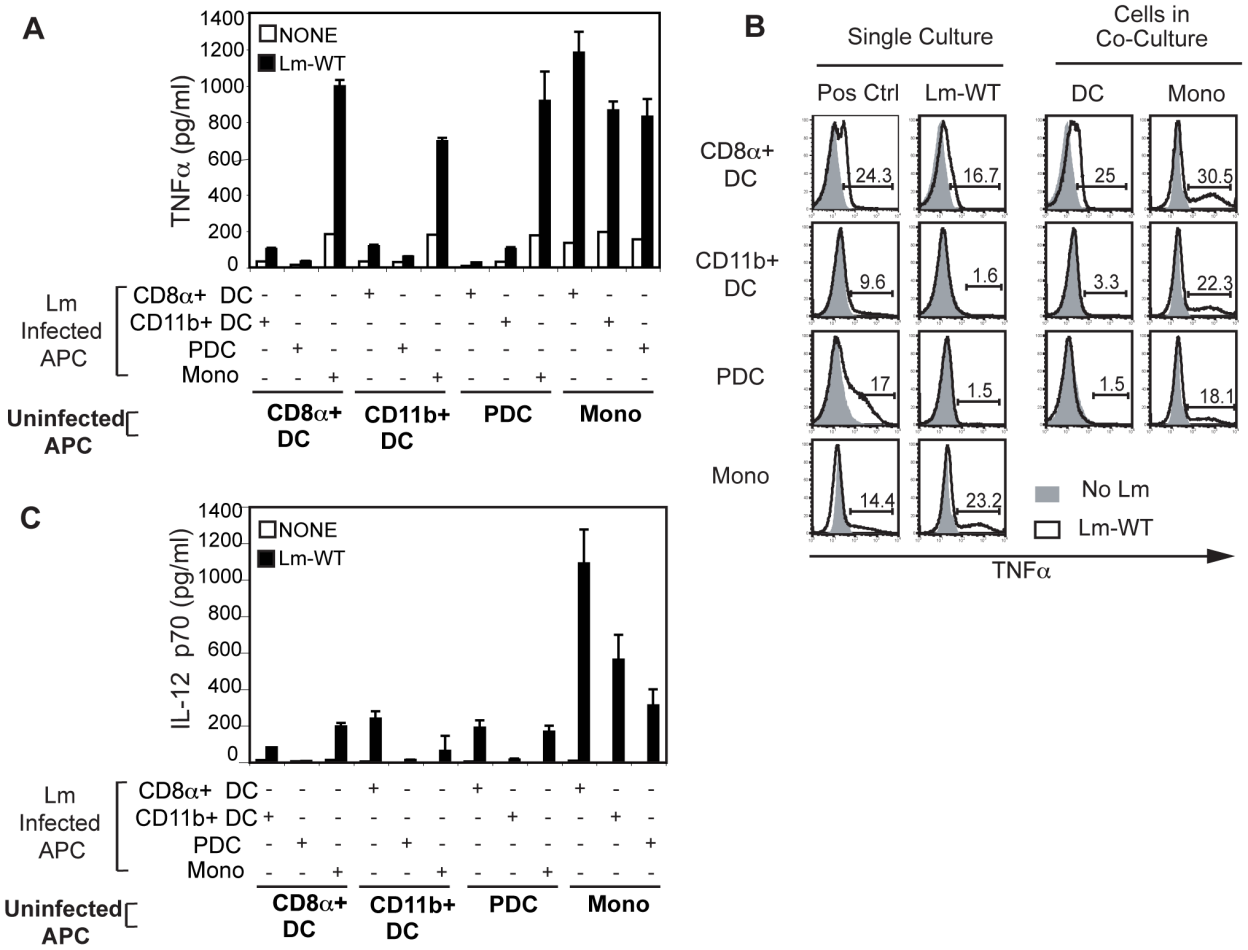
TNFα and IL-12 p70 from infected DC subsets is further increased with co-culture with monocytes. Sorted APCs were infected with Lm-WT and incubated overnight in gentamicin media at 37°C. Infected subsets were co-cultured with uninfected sorted APCs (**bold labels**) as indicated. Supernatants from the co-cultures were assayed for (**A**) TNFα and (**C**) IL-12 p70 by a Luminex-based multiplex cytokine assay. Data are representative of at least three independent experiments. (**B**) For determination of TNFα secretion via ICS, Lm-infected DCs or monocytes or co-cultures were incubated in gentamicin media for 16 hrs. Cultures were treated with brefeldin A for the final 12 hours at 37°C, and TNFα secretion was assessed via flow cytometry. Data were gated on either CD11c^hi^ CD11b^−^, CD11c^hi^ CD11b^+^, CD11c^int^ PDCA-1^+^ or CD11c^−^ CD11b^+^ events, and the percentage of cells positive for TNFα was determined using FlowJo software. (Shaded histograms represent uninfected cells while empty histograms with solid line represent Lm-WT infected cells). Data for CD11b^+^ DCs cultured with monocytes are shown here and are representative of two independent experiments.

We next asked if co-culture of different DC subsets with monocytes would modulate TNFα and IL-12 p70 secretion from these DCs. DC subsets and monocytes were infected as described before, washed and then incubated in gentamicin-containing media with other uninfected DC subsets or monocytes. While a significant increase in levels of TNFα was readily detectible from co-cultures containing infected monocytes (Figure 3A), levels of TNFα from infected DC co-cultures were much lower. No enhancement of TNFα secretion was observed due to collaboration between DC subsets. Co-cultures between infected DCs and uninfected monocytes resulted in significant increases in cytokine secretion even in the case of infected PDCs, and levels of TNFα measured were comparable to those from infected monocytes. To identify the source of cytokine production by ICS, we found that both the infected DC and the monocytes, which were not directly infected, produce TNFα (Figure 3B).

With regards to IL-12 p70, we saw enhanced secretion when infected DCs were co-cultured with uninfected monocytes (Figure 3C). While levels of IL-12 p70 from cultures including infected CD8α^+^ DCs or monocytes alone were low, we observed a 5–10 fold enhancement in co-cultures between each of the infected DC subsets and uninfected monocytes. Maximal levels of IL-12 p70 were observed in co-cultures between CD8α^+^ DCs and uninfected monocytes, but modest increases were also measured from infected CD11b+ DCs and PDCs, which could result from maturation of these monocytes into TipDC-like cells.

### Interaction between DCs and monocytes during Lm infection *ex vivo* abrogates T cell priming

**Figure 4:**
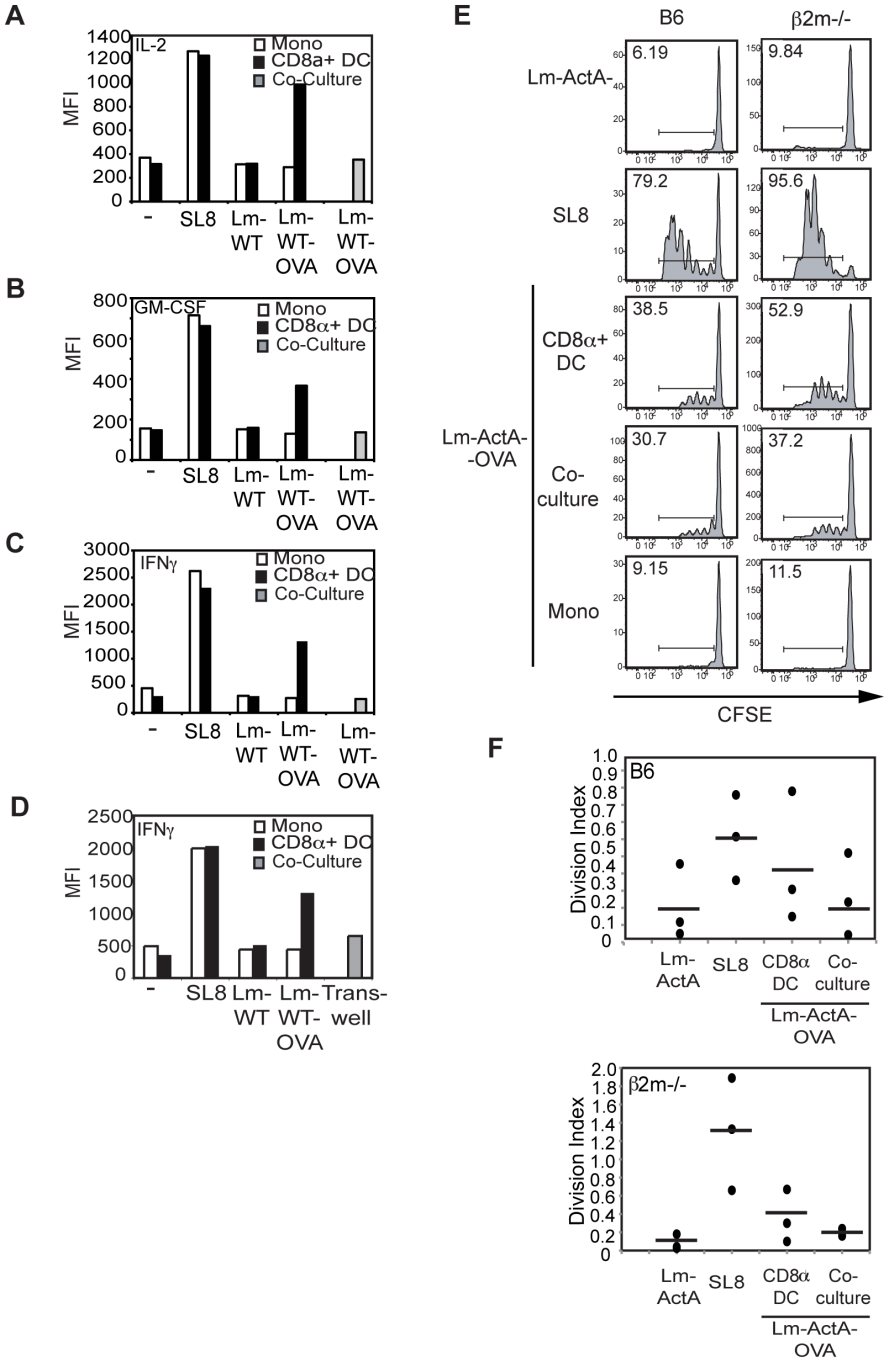
Priming of T cells by APC co-cultures is attenuated *in vitro* and *in vivo*. Sorted CD8α^+^ DCs and monocytes were infected with Lm-WT or Lm-WT-OVA at an MOI of 1 either individually or together in co-cultures. 10^4^ infected DCs or monocytes were then cultured at 37°C with 10^5^ purified OT-1 transgenic T cells. On Day 4 T cells were washed and re-stimulated with B6 total splenocytes pulsed with SL8 peptide for 5 hours in the presence of brefeldin A. Production of (**A**) IL-2, (**B**) GM-CSF and (**C**) IFNγ was determined via ICS, and MFI of cytokine staining was analyzed using FlowJo software. (**D**) Lm-WT or Lm-WT-OVA infected CD8α^+^ DCs or monocytes were then cultured at 37°C with purified OT-1 transgenic T cells in the bottom of a trans-well chamber. In the case of APC co-cultures, infected CD8α^+^ DCs were cultured with the T cells in the bottom of a trans-well chamber while infected monocytes were seeded in the top of the trans-well chamber. IFNγ secretion was assessed via ICS and MFI of cytokine staining was determined using FlowJo software. (**E**) CD8^+^ OT1 T cells were isolated and labeled with 5 mM CFSE. 1×10^6^ cells were adoptively transferred intravenously (i.v.) into either wild-type B6 (left column) or β2m^−/−^ (right column) mice. Sorted CD8α^+^ DCs or monocytes infected with either Lm-actA^−^, Lm-actA^−^-OVA or pulsed with 1 mM SL8 peptide were injected subcutaneously (s.c.) into the foot-pad 8 hours later. Popliteal lymph nodes were harvested at 60 hrs. CFSE dilution and cell division were determined using FlowJo software. Numbers in histograms represent percent divided cells. Data shown in panels (A–E) are for one mouse per condition and are representative of at least three independent experiments. (**F**) The division indices for mice from 3 separate experiments (circles) are shown along with the averages for each condition (dashes). Analysis was performed using the Proliferation algorithm in the Flowjo software.

Given the marked enhancement in IL-12 p70 secretion in the presence of monocytes, we studied the effect of this interaction between CD8α^+^ DCs and monocytes on T cell activation. Having determined that CD8α^+^ DCs were not just the most efficient but the only DC subsets capable of inducing T cell priming and CTL differentiation, we chose to focus on this subset for further studies. CD8α^+^ DCs infected with Lm-WT-OVA were co-cultured with monocytes, and OVA-reactive OT1^+^ T cells. IL-2, GM-CSF and IFNγ production were assayed by ICS on D4. Co-cultures between CD8α^+^ DC and monocytes did not enhance T cell activation (data not shown) or IFNγ secretion. Surprisingly, direct contact with infected monocytes abrogated IL-2, GM-CSF and IFNγ secretion from OT1 T cells (Figure 4A, B, C). Interestingly, co-culture with uninfected monocytes did not result in abrogation, suggesting that infection of monocytes was required for inhibition (data not shown). In addition, this inhibition was also apparent in trans-well cultures (Figure 4 D).

To test whether a similar effect is seen *in vivo*, we immunized mice with CD8α^+^ DC and monocytes that were co-infected *ex vivo* and assessed T cell responses via CFSE dilution. Co-immunization with infected CD8α^+^ DC and monocytes resulted in abrogated T cell proliferation *in vivo* (Figure 4E, left column). To assess the role of cross-presentation in priming this immune response, we also repeated these studies in beta2-microglobulin deficient (β2m^−/−^) mice [38], [39]. Responses to Lm in β2m^−/−^ mice mimicked the results seen in wild-type mice, and infected CD8α^+^ DCs were able to elicit a response (Figure 4E, right column). Since these mice are unable to cross-present Lm-derived antigens on MHC I, we can assume the T cell activation elicited is a direct response to *ex vivo* infected DCs and not to other host-derived cells. Consistent with our *in vitro* data, we also observed a decrease in T cell priming with co-administration of CD8α^+^ DC and monocyte (Figure 4E). This effect was particularly evident in β2m^−/−^ recipient mice. We also analyzed the average number of cell divisions in these populations using the division index based upon CSFE dye dilution (Figure 4F). The division index represents the average number of cell divisions of all cells in the population (i.e. it includes the undivided cells). Once again, the data revealed that the average number of T cell divisions in co-cultures in both B6 mice (top panel) and β2m^−/−^ mice (bottom panel) is decreased modestly compared to T cell cultures with infected CD8α^+^ DCs only. These combined data suggest that antigen specific T cells can be primed via direct presentation and that cross-presentation may not necessarily be the primary mechanism for T cell activation in response to Lm *in vivo*. In addition, the magnitude of the response in β2m^−/−^ mice was somewhat greater compared to that in the wild-type mice. We speculate that the increase may be due to differences in the cytokine milieu or stimulatory signals yet to be elucidated in the β2m^−/−^ mice.

**Figure 5:**
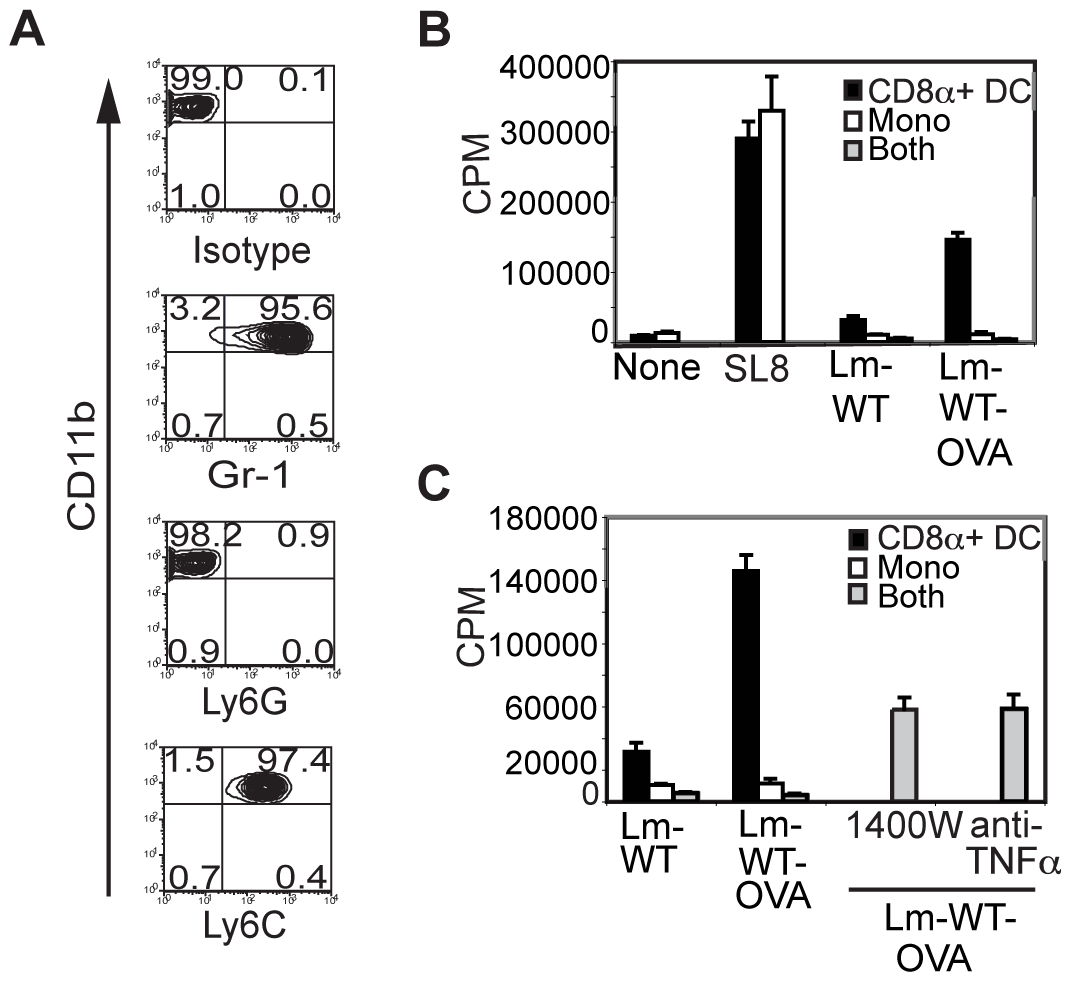
Inhibition of T cell priming by Gr-1^+^Ly6C^+^ monocytes is mediated partly by TNFα and iNOS. (**A**) Sorted monocytes were stained with antibodies against Gr-1, Ly6C and Ly6G and assessed by flow cytometry. Dot plots are gated on CD11c^−^CD11b^+^ cells and numbers in corners represent percentages of cells staining positive for the given marker. Data are representative of two independent experiments. (**B**) CD8α^+^ DCs and monocytes were infected with Lm-WT or Lm-WT-OVA either individually or as a co-culture as described, and 5×10^4^ APCs were incubated in gentamicin containing media with 10^5^ purified CD8^+^ OT1^+^ T cells at a ratio of 2∶1 (T∶DC, or T∶mono; final cell count 1.5×10^5^) or 2∶1∶1 (T∶DC∶mono; final cell count 2×10^5^) for 60–70 hrs. T cell proliferation was assessed with the addition of ^3^H-thymidine for the final 16 hrs of incubation. (**C**) To determine the role of TNFα and iNOS, samples infected as described in **panel B** were incubated either in the presence or absence of 10 µg/ml of a neutralizing monoclonal antibody against TNFα or 1400W, an inhibitor of iNOS. Data are representative of two independent experiments.

With these observed suppressive effects, we phenotypically characterized the sorted splenic monocytes (CD11c^−^CD11b^+^ cells). The monocytes isolated from Flt3-L mobilized spleens for our study were Ly6C^+^ Gr-1^+^ Ly6G^−^ (Figure 5A), a phenotype consistent with TipDCs [6], [40]. This finding suggests that the sorted splenic monocytes may represent the cells that develop into TipDCs with Lm infection [6]. To examine whether either TNFα or nitric oxide (NO) mediates the observed inhibition, we incubated infected CD8α^+^ DCs and monocytes with OT1^+^ CD8^+^ T cells in the presence of TNFα or iNOS inhibitors. Consistent with our prior results, the addition of monocytes to CD8α^+^ DCs/T cell cultures dampened T cell proliferation (Figure 5B). The addition of 1400W, an iNOS inhibitor, or a neutralizing monoclonal antibody to TNFα resulted in a partial but significant release from inhibition indicating that both these molecules are involved in mediating T cell inhibition (Figure 5C). There was no additive effect of blocking both inhibitors together (data not shown). While a direct effect of these mediators on CD8α^+^ DC cannot be excluded at this point, we hypothesize that it is likely that these compounds mediate a more significant impact on the co-cultured monocytes. While these molecules have been implicated in bacterial clearance by TipDCs, it is surprising to find that both these molecules can inhibit T cell activation. While TNFα can contribute to the pro-inflammatory environment, this cytokine my also lead to the terminal activation of antigen presenting cells, perhaps contributing to increased apoptosis of the presenting DCs and decreased persistence of antigen. These data also highlight the delicate counterbalance between the simultaneous induction of both stimulatory and inhibitory pathways as a result of microbial infection.

## Discussion

In this study, we assessed the contributions of different DC subsets and monocytes in generating an immune response against the intracellular bacterium Lm. The spleen is a known site of Lm infection. By infecting isolated splenic APCs *ex vivo* in the absence of other cell types, we could dissect the specific contributions of each subset and demonstrate that monocytes can influence CD8α^+^ DC function and dampen the subsequent adaptive T cell responses. We also find that PDCs are strikingly refractory to infection *in vitro*. In view of studies that have shown the detrimental effects of type I IFNs on bacterial clearance [15], [16], [17], this lack of participation in this anti-bacterial response may reflect the checks and balances inherent in the host immune system to maintain a productive response while limiting infection-induced immune-mediated pathology. Nevertheless, one study has described the activation of PDCs in response to intragastric infection with Lm *in vivo* [41], and these data suggest a role for PDCs at the site of bacterial entry that may require accessory cells or environmental cues for involvement in the response.

The monocytes isolated for our *ex vivo* experiments exhibited low levels of infection but secrete high levels of inflammatory cytokines (TNFα and IL-12 p70) in our hands, signature cytokines secreted by classically activated macrophages [31]. Functional IL-12 p70 from monocytes has been reported to be produced by human monocytes [42]. In addition, maximal cytokine secretion from infected DCs (especially IL-12 p70 from CD8α^+^ DCs) is observed when these DCs are co-cultured with monocytes. These findings are in keeping with recent evidence highlighting the interplay between innate mediators such as TipDCs early in an Lm-specific response [40]. The monocytes isolated in our study are also Gr-1^+^Ly6C^+^Ly6G^−^ and appear to be functionally similar to TipDCs as they are able to secrete TNFα while appearing uninfected [6].

Our study also emphasizes the role of Lm-exposed monocytes as important modulators of the adaptive response to Lm. The enhanced production of IL-12 seen would presumably enhance priming of antigen specific CD8+ T cells. Nevertheless, the capacity of CD8α^+^ DCs to prime T cells and elicit T cell effector function efficiently is in fact attenuated *ex vivo* and *in vivo* in the presence of infected monocytes. These data are consistent with several *in vivo* studies that have shown that T cell responses are abrogated during the early stages of the Lm response [43], [44]. Moreover, antigen specific responses to Lm in the spleens of CCR2 deficient mice that are unable to recruit TipDCs are enhanced compared to those in wild-type infected spleens [6]. We also demonstrate that this inhibition of T cell activation was at least in part due to the effects of iNOS and TNFα, two mediators essential for antimicrobial defenses *in vivo*. While monocytes would be the presumed source of iNOS, DCs may also represent another source [45], [46].

Thus, Lm-exposed monocytes exhibit distinct characteristics of both TipDC like precursors and inflammatory monocytes and may represent a unique subset capable of both enhancing and abrogating different components of Lm-specific responses. During Lm infection, monocytes may sense Lm infected DCs under inflammatory conditions via some soluble mediator including elaborated cytokines or chemokines such as monocyte chemoattractant protein-1 (MCP-1). Such Lm-exposed monocytes then differentiate into TipDCs to secrete TNFα and NO. While important for elaborating inflammatory cytokines crucial for bacterial clearance and the innate immune response, these TipDC like inflammatory monocytes appear to unexpectedly abrogate adaptive responses. This paradoxical augmentation of DC cytokine secretion but impairment in T cell stimulation could be explained in light of the competing goals of clearing vs. establishing durable protective immunity. CD8α^+^ DCs being the primary splenic DC subset capable of cross-presentation are uniquely situated to mount a potent immune response against infectious pathogens. However in the case of Lm, these APCs are also highly susceptible to infection, making them a liability to the host as a conduit for disseminating the infection. Dampening an immune response may be desirable to restrict tissue damage as a result of unregulated release of microbicidal mediators such as TNFα and NO. Alternatively, impairment of T cell responses to infected DCs may represent an as yet unappreciated mechanism by which Lm subverts the host to avoid detection and preserve its intracellular niche.

## Materials and Methods

### Mice

Mice were purchased from the NIH/National Cancer Institute (NCI), Charles River (Wilmington, MA) or Jackson Labs (Bar Harbor, ME). Mice used in this study were wild-type C57BL/6 (B6) mice, OT1 TCR transgenic mice [37], and beta 2-microglobulin deficient (β2m^−/−^) mice [38], [39]. Mice were housed in a specific pathogen free facility and experiments were performed in an appropriate biosafety level 2 (BSL2) facility as per UCSF protocol.

### Bacterial strains

*Listeria monocytogenes* strains used were the wild-type Lm-WT (DP-L4056), and the Lm-ActA^−^ (DP-L4029 Δ*actA*) mutant strain, in addition to the isogenic strains containing the pPL2-LLO-OVA cassette Lm-WT pPL2-LLO-OVA (Lm-WT-OVA) and Lm-ActA^−^ pPL2-LLO-OVA (Lm-ActA^−^-OVA) (Aduro Biotech, Berkeley, CA). The *in vivo* 50% lethal dose [LD_50_] in B6 mice of the wild-type strain is 5×10^4^ colony forming units (cfu) and the Lm-ActA^−^ strain is 1×10^8^ cfu, and have been described previously [47].

### Peptides and Antibodies

OVA_257–264_ (SIINFEKL; SL8) peptide was synthesized by Synpep Corporation (Dublin, CA). Antibodies used for DC isolation were: CD11b FITC (clone M1/70; BioLegend, San Diego, CA), PDCA-1 PE (Clone JF05-1C2.4.1; Miltenyi Biotec Inc., Auburn, CA), PDCA-1 APC (Clone eBio927; eBiosciences, San Diego, CA) and CD11c Alexa 647 (clone N418; home grown). Antibodies used in this study were: CD40 PE/Cy5 (clone 1C10), MHCII PE, (Clone M5/114.15.2), Vα2^+^ TCR FITC (clone B20.1), CD11c PE (clone HL3), CD4 PE (clone GK1.5), CD11b PE (clone M1/70), CD8α PerCP (clone 53-6.7), CD25 APC (clone PC61), Ly6C PE (clone AL-21), Ly6G PE (clone 1A8) from BD Pharmingen (San Jose, CA); CD80 PE (clone 16-10A1), CD86 Pacific Blue (clone GL1), CD44 Pacific Blue (clone IM7), IL-2 Pacific Blue (clone JES6-5H4), GMCSF PE (clone MP1-22E9), Gr-1 PE (clone RB6 8C5), Streptavidin PE-Cy7 from BioLegend (San Diego, CA); CD62L PE (clone MEL 14), CD69 PE-Cy7 (clone H1.2F3), IFNγ APC (clone XMG1.2), TNFα PE (clone MP6-XT22), from eBiosciences (San Diego, CA).

### Dendritic cell isolation

B6 Mice were injected subcutaneously in the right flank with 5×10^6^ B16-BL6 melanoma tumor cells engineered to secrete FMS like Tyrosine kinase 3 ligand (Flt3-L) [48]. Spleens were harvested between d12-d16, injected with 2 mg/ml Collagenase D (Roche, San Francisco, CA) and incubated at 37°C for 20 minutes. Spleens were then disrupted with the flat end of a syringe plunger, washed and treated with Tris ammonium chloride buffer (TAC) to lyse the red blood cells. Splenocytes were then stained with CD11b FITC, PDCA-1 PE and CD11c Alexa 647, and DC subsets were sorted on a MoFlo sorter (DakoCytomation, Fort Collins, CO) as CD11c^hi^ CD11b^−^ PDCA-1^−^ (CD8α^+^ DC), CD11c^hi^ CD11b^+^ PDCA-1^−^ (CD11b^+^ DC), CD11c ^int^ CD11b^−^ PDCA-1^+^ (PDC). Monocytes were sorted as the CD11c^−^ CD11b^+^ PDCA-1^−^ subset.

### Bacterial infections

Overnight cultures or log phase cultures of Lm strains were grown in brain-heart infusion (BHI) media (Difco-BD Biosciences, CA) and used for infection as previously described [49]. Briefly, sorted cells were incubated with Lm at a multiplicity of infection (MOI) of 1. Samples were infected while rolling for 1 hr at 37°C and then washed twice in media containing 50 µg/ml gentamicin (Sigma-Aldrich, St. Louis, MO) to prevent growth of extracellular bacteria. The infected cells were then incubated in gentamicin media for the indicated length of time and assayed as described below.

### Colony forming unit (CFU) determination

Sorted DC subsets were infected at an MOI of 1 and incubated for 1 hr at 37°C. Following infection, the cells were washed and incubated in media containing 50 µg/ml gentamicin (Sigma-Aldrich, St. Louis, MO) for approximately 1 hr at 37°C to kill extracellular bacteria. The infected cells were then lysed in 0.5% NP40 (Sigma-Aldrich, St. Louis, MO) and plated on BHI agar plates. The plates were incubated overnight at 37°C and Lm colonies were enumerated after 24–48 hrs as a measure of infection for each DC subset.

### B3Z T cell hybridoma assay

The B3Z T cell hybridoma is an OVA _257–264_ (SIINFEKL; SL8)/H-2K^b^ specific CD8^+^ T cell hybridoma that is *LacZ*-inducible [36]. DC subsets infected as described were washed and co-incubated with B3Z cells at a ratio of 1∶1 in gentamicin containing media. Cultures were incubated for 20–24 hours at 37°C and developed with CPRG substrate (CalBiochem, San Diego, CA) for 2–4 hr at 37°C as described [36].

### Assessment of cytokine responses

Infected DC subsets were cultured in gentamicin containing media for 18–20 hrs in the presence or absence of 100 U/mL IFNγ (R&D, Systems, Minneapolis, MN). Supernatants were harvested and assayed for IFNα using the murine IFNα ELISA kit (PBL Biomedical Laboratories, Piscataway, NJ); or TNFα, IL-12 p40 and IL-12 p70 using multiplex cytokine assay kits (Bio-Rad; Hercules, CA) as per the manufacturer's protocol. Multiplex cytokine assays were analyzed on a Luminex100 (Luminex; Austin, TX). TNFα and IL-12 p70 were also measured using ELISA kits from R&D Systems (Minneapolis, MN) and e-Bioscience (San Diego, CA) as per the manufacturer's protocols. Alternatively for determination of TNFα secretion via intracellular cytokine staining (ICS), Lm-infected DCs or monocytes or co-cultures were incubated in gentamicin media for 16 hrs. Cultures were treated with brefeldin A for the final 12 hours at 37°C and fixed and permeabilized with BD cytofix/cytoperm buffers (BD Biosciences, San Jose, CA). Cells were stained with antibodies specific for TNFα, acquired on a FACS LSRII (BD Biosciences, San Jose, CA), and gated on either CD11c^hi^ CD11b^−^, CD11c^hi^ CD11b^+^, CD11c^int^ PDCA-1^+^ or CD11c^−^ CD11b^+^ cells. The percentage of cells positive for TNFα and geometric mean fluorescence of staining was determined using FlowJo software v6.6 (Treestar, Ashland, OR).

### Intracellular staining for T cell activation

CD8^+^ OT1 T cells were isolated by negative or positive selection from splenocytes of OT1 T cell receptor transgenic mice [37] using a CD8^+^T cell isolation kit (either Miltenyi Biotec Inc., Auburn, CA or StemCell Technologies, Vancouver, BC, Canada). OT1 CD8^+^ T cells were routinely found to be 90–95% pure. 10^4^ Lm infected DCs or monocytes were co-incubated with 10^5^ OT1 T cells in gentamicin media at 37°C for 4 days. Cells in culture were at a final ratio of 10∶1 (T∶DC, T∶mono; final cell count 1.1×10^5^) or 10∶1∶1 (T∶DC∶mono; final cell count 1.2×10^5^). For trans-well experiments, monocytes in the co-culture wells (T+DC+Mono) were seeded in the upper chamber of the trans-well while T cells and DCs were cultured in the lower chamber. Where DCs alone or monocytes alone were cultured with OT1 T cells the APCs were seeded in the lower chamber with the T cells. On D4 cells were washed and stimulated with splenocytes from B6 mice pulsed with 50 µg/ml SL8 peptide in the presence of brefeldin A for 5 hours at 37°C. The cells were stained for Vα2 and CD8, and fixed and permeabilized with BD cytofix/cytoperm buffers (BD Biosciences, San Jose, CA). Cells were stained with antibodies specific for IL-2, GM-CSF and IFNγ and acquired on a FACS LSRII (BD Biosciences, San Jose, CA). Data were gated on Vα2^+^ CD8^+^ events, and the percentage of cells positive for IL-2, GM-CSF or IFNγ and geometric mean fluorescence of staining was determined using FlowJo software v6.6 (Treestar, Ashland, OR).

### *In vivo* T cell activation

CD8^+^ OT1 T cells were isolated as described above and labeled with 5 mM carboxyfluorescein diacetate succinimidyl ester (CFSE; Molecular Probes, Eugene, OR). 1×10^6^ cells were injected intravenously (i.v.) into either naïve B6 or β2m^−/−^ mice. Sorted DC subsets infected with either Lm-ActA^−^, Lm-ActA^−^-OVA or pulsed with 1 mM SL8 peptide were injected subcutaneously in the foot-pads of these mice 8 hours later. Popliteal lymph nodes were harvested at 60 hrs, and T cell proliferation was determined via CFSE dilution. The division index of proliferated cultures was derived by the formula: ***Division Index*** *= (* ***Proliferation Index*** *)* ***×*** *(* ***Percent Divided*** *)*, where the proliferation index is the average number of cell divisions of the responder cells. Samples were acquired on a BD FACS LSRII (BD Biosciences, San Jose, CA) and data were gated on Vα2^+^ CD8^+^ events. The percentages of activated cells and CFSE analyses were determined using FlowJo software (Treestar, Ashland, OR).

### *In vitro* T cell proliferation

CD8^+^ OT1 T cells were purified as described. CD8α^+^ DCs and monocytes were infected with Lm-WT or Lm-WT-OVA either individually or as a co-culture as described and 5×10^4^ APCs were cultured with 10^5^ purified OT1^+^ T cells for 60–70 hrs. Cells in culture were at a ratio of 2∶1 (T∶DC or T∶mono; final cell count 1.5×10^5^) or 2∶1∶1 (T∶DC∶mono; final cell count 2×10^5^). T cell proliferation was assessed with the addition of ^3^H-thymidine (PerkinElmer, Boston, MA) for the final 16 hrs of incubation. To determine the role of TNFα and iNOS, samples were incubated either in the presence or absence of 10 µg/ml of a monoclonal antibody against TNFα (clone MP6-XT22, Biolegend, San Diego, CA) or 1400W (Sigma-Aldrich, St. Louis, MO), an inhibitor of iNOS. Cultures were then harvested using a Tomtec Harvester 96 (Hamden, CT) and counted on a Wallac 1450 Microbeta Trilux liquid scintillation counter (EG&G Wallac, Turku, Finland) using Wallac Software version 2.7.

## Supporting Information

Figure S1 **Phenotype of DCs from Flt3-L mobilized mice and DCs from unmobilized mice is similar.** Total splenocytes isolated from Flt3-L mobilized mice and from wild-type mice were stained with antibodies against CD80, CD86, CD40 and MHC II. Cells were gated on DC subsets identified using the gating strategy described in **Figure 1 A**. Expression levels of these markers were determined using FlowJo software. Shaded histograms represent isotype control staining while empty histograms with solid line represent activation marker specific staining. Numbers over gates represent cell frequency.
(TIF)

## References

[1] Pamer EG (2004). Immune responses to Listeria monocytogenes.. Nat Rev Immunol.

[2] Zenewicz LA, Shen H (2007). Innate and adaptive immune responses to Listeria monocytogenes: a short overview.. Microbes Infect.

[3] Neuenhahn M, Busch DH (2007). Unique functions of splenic CD8alpha+ dendritic cells during infection with intracellular pathogens.. Immunol Lett.

[4] Cossart P, Toledo-Arana A (2008). Listeria monocytogenes, a unique model in infection biology: an overview.. Microbes Infect.

[5] Serbina NV, Jia T, Hohl TM, Pamer EG (2008). Monocyte-mediated defense against microbial pathogens.. Annu Rev Immunol.

[6] Serbina NV, Salazar-Mather TP, Biron CA, Kuziel WA, Pamer EG (2003). TNF/iNOS-producing dendritic cells mediate innate immune defense against bacterial infection.. Immunity.

[7] Conlan JW, North RJ (1994). Neutrophils are essential for early anti-Listeria defense in the liver, but not in the spleen or peritoneal cavity, as revealed by a granulocyte-depleting monoclonal antibody.. J Exp Med.

[8] Rogers HW, Unanue ER (1993). Neutrophils are involved in acute, nonspecific resistance to Listeria monocytogenes in mice.. Infect Immun.

[9] Unanue ER (1997). Inter-relationship among macrophages, natural killer cells and neutrophils in early stages of Listeria resistance.. Curr Opin Immunol.

[10] Czuprynski CJ, Brown JF, Wagner RD, Steinberg H (1994). Administration of antigranulocyte monoclonal antibody RB6-8C5 prevents expression of acquired resistance to Listeria monocytogenes infection in previously immunized mice.. Infect Immun.

[11] Dunn PL, North RJ (1991). Early gamma interferon production by natural killer cells is important in defense against murine listeriosis.. Infect Immun.

[12] Huang S, Hendriks W, Althage A, Hemmi S, Bluethmann H (1993). Immune response in mice that lack the interferon-gamma receptor.. Science.

[13] Rothe J, Lesslauer W, Lotscher H, Lang Y, Koebel P (1993). Mice lacking the tumour necrosis factor receptor 1 are resistant to TNF-mediated toxicity but highly susceptible to infection by Listeria monocytogenes.. Nature.

[14] Pfeffer K, Matsuyama T, Kundig TM, Wakeham A, Kishihara K (1993). Mice deficient for the 55 kd tumor necrosis factor receptor are resistant to endotoxic shock, yet succumb to L. monocytogenes infection.. Cell.

[15] O'Connell RM, Saha SK, Vaidya SA, Bruhn KW, Miranda GA (2004). Type I interferon production enhances susceptibility to Listeria monocytogenes infection.. J Exp Med.

[16] Carrero JA, Calderon B, Unanue ER (2004). Type I interferon sensitizes lymphocytes to apoptosis and reduces resistance to Listeria infection.. J Exp Med.

[17] Auerbuch V, Brockstedt DG, Meyer-Morse N, O'Riordan M, Portnoy DA (2004). Mice lacking the type I interferon receptor are resistant to Listeria monocytogenes.. J Exp Med.

[18] Conlan JW (1996). Early pathogenesis of Listeria monocytogenes infection in the mouse spleen.. J Med Microbiol.

[19] Steinman RM, Hemmi H (2006). Dendritic cells: translating innate to adaptive immunity.. Curr Top Microbiol Immunol.

[20] Steinman RM (2007). Dendritic cells: understanding immunogenicity.. Eur J Immunol.

[21] Shortman K, Liu YJ (2002). Mouse and human dendritic cell subtypes.. Nat Rev Immunol.

[22] Shortman K, Naik SH (2007). Steady-state and inflammatory dendritic-cell development.. Nat Rev Immunol.

[23] Jung S, Unutmaz D, Wong P, Sano G, De los Santos K (2002). In vivo depletion of CD11c(+) dendritic cells abrogates priming of CD8(+) T cells by exogenous cell-associated antigens.. Immunity.

[24] Pron B, Boumaila C, Jaubert F, Berche P, Milon G (2001). Dendritic cells are early cellular targets of Listeria monocytogenes after intestinal delivery and are involved in bacterial spread in the host.. Cell Microbiol.

[25] Neuenhahn M, Kerksiek KM, Nauerth M, Suhre MH, Schiemann M (2006). CD8alpha+ dendritic cells are required for efficient entry of Listeria monocytogenes into the spleen.. Immunity.

[26] Belz GT, Shortman K, Bevan MJ, Heath WR (2005). CD8alpha+ dendritic cells selectively present MHC class I-restricted noncytolytic viral and intracellular bacterial antigens in vivo.. J Immunol.

[27] Iyoda T, Shimoyama S, Liu K, Omatsu Y, Akiyama Y (2002). The CD8+ dendritic cell subset selectively endocytoses dying cells in culture and in vivo.. J Exp Med.

[28] Kim TS, Braciale TJ (2009). Respiratory dendritic cell subsets differ in their capacity to support the induction of virus-specific cytotoxic CD8+ T cell responses.. PLoS One.

[29] Bedoui S, Whitney PG, Waithman J, Eidsmo L, Wakim L (2009). Cross-presentation of viral and self antigens by skin-derived CD103+ dendritic cells.. Nat Immunol.

[30] Le Borgne M, Etchart N, Goubier A, Lira SA, Sirard JC (2006). Dendritic cells rapidly recruited into epithelial tissues via CCR6/CCL20 are responsible for CD8+ T cell crosspriming in vivo.. Immunity.

[31] Mosser DM, Edwards JP (2008). Exploring the full spectrum of macrophage activation.. Nat Rev Immunol.

[32] Torres D, Barrier M, Bihl F, Quesniaux VJ, Maillet I (2004). Toll-like receptor 2 is required for optimal control of Listeria monocytogenes infection.. Infect Immun.

[33] Humann J, Lenz LL (2010). Activation of naive NK cells in response to Listeria monocytogenes requires IL-18 and contact with infected dendritic cells.. J Immunol.

[34] Mempel TR, Henrickson SE, Von Andrian UH (2004). T-cell priming by dendritic cells in lymph nodes occurs in three distinct phases.. Nature.

[35] Mora JR, Bono MR, Manjunath N, Weninger W, Cavanagh LL (2003). Selective imprinting of gut-homing T cells by Peyer's patch dendritic cells.. Nature.

[36] Sanderson S, Shastri N (1994). LacZ inducible, antigen/MHC-specific T cell hybrids.. Int Immunol.

[37] Hogquist KA, Jameson SC, Heath WR, Howard JL, Bevan MJ (1994). T cell receptor antagonist peptides induce positive selection.. Cell.

[38] Zijlstra M, Bix M, Simister NE, Loring JM, Raulet DH (1990). Beta2-Microglobulin deficient mice lack CD4-8+ cytolytic T cells.. Nature.

[39] Koller BH, Marrack P, Kappler JW, Smithies O (1990). Normal development of mice deficient in beta 2M, MHC class I proteins, and CD8+ T cells.. Science.

[40] Kang SJ, Liang HE, Reizis B, Locksley RM (2008). Regulation of hierarchical clustering and activation of innate immune cells by dendritic cells.. Immunity.

[41] Tam MA, Wick MJ (2006). Differential expansion, activation and effector functions of conventional and plasmacytoid dendritic cells in mouse tissues transiently infected with Listeria monocytogenes.. Cell Microbiol.

[42] Tada Y, Asahina A, Takekoshi T, Kishimoto E, Mitsui H (2006). Interleukin 12 production by monocytes from patients with psoriasis and its inhibition by ciclosporin A.. Br J Dermatol.

[43] Jiang J, Lau LL, Shen H (2003). Selective depletion of nonspecific T cells during the early stage of immune responses to infection.. J Immunol.

[44] Merrick JC, Edelson BT, Bhardwaj V, Swanson PE, Unanue ER (1997). Lymphocyte apoptosis during early phase of Listeria infection in mice.. Am J Pathol.

[45] Lu L, Bonham CA, Chambers FG, Watkins SC, Hoffman RA (1996). Induction of nitric oxide synthase in mouse dendritic cells by IFN-gamma, endotoxin, and interaction with allogeneic T cells: nitric oxide production is associated with dendritic cell apoptosis.. J Immunol.

[46] Ren G, Su J, Zhao X, Zhang L, Zhang J (2008). Apoptotic cells induce immunosuppression through dendritic cells: critical roles of IFN-gamma and nitric oxide.. J Immunol.

[47] Brockstedt DG, Giedlin MA, Leong ML, Bahjat KS, Gao Y (2004). Listeria-based cancer vaccines that segregate immunogenicity from toxicity.. Proc Natl Acad Sci U S A.

[48] Dranoff G, Jaffee E, Lazenby A, Golumbek P, Levitsky H (1993). Vaccination with irradiated tumor cells engineered to secrete GM-CSF stimulates potent, specific, and long lasting anti-tumor immunity.. Proc Natl Acad Sci USA.

[49] Brockstedt DG, Bahjat KS, Giedlin MA, Liu W, Leong M (2005). Killed but metabolically active microbes: a new vaccine paradigm for eliciting effector T-cell responses and protective immunity.. Nat Med.

