# Supplementary figures and images for: Interplay between CD8α+ Dendritic Cells and Monocytes in Response to Listeria monocytogenes Infection Attenuates T Cell Responses
Source: PLoS One. 2011 Apr 29;6(4):e19376. doi: 10.1371/journal.pone.0019376 (PMC3084837; doi:10.1371/journal.pone.0019376)

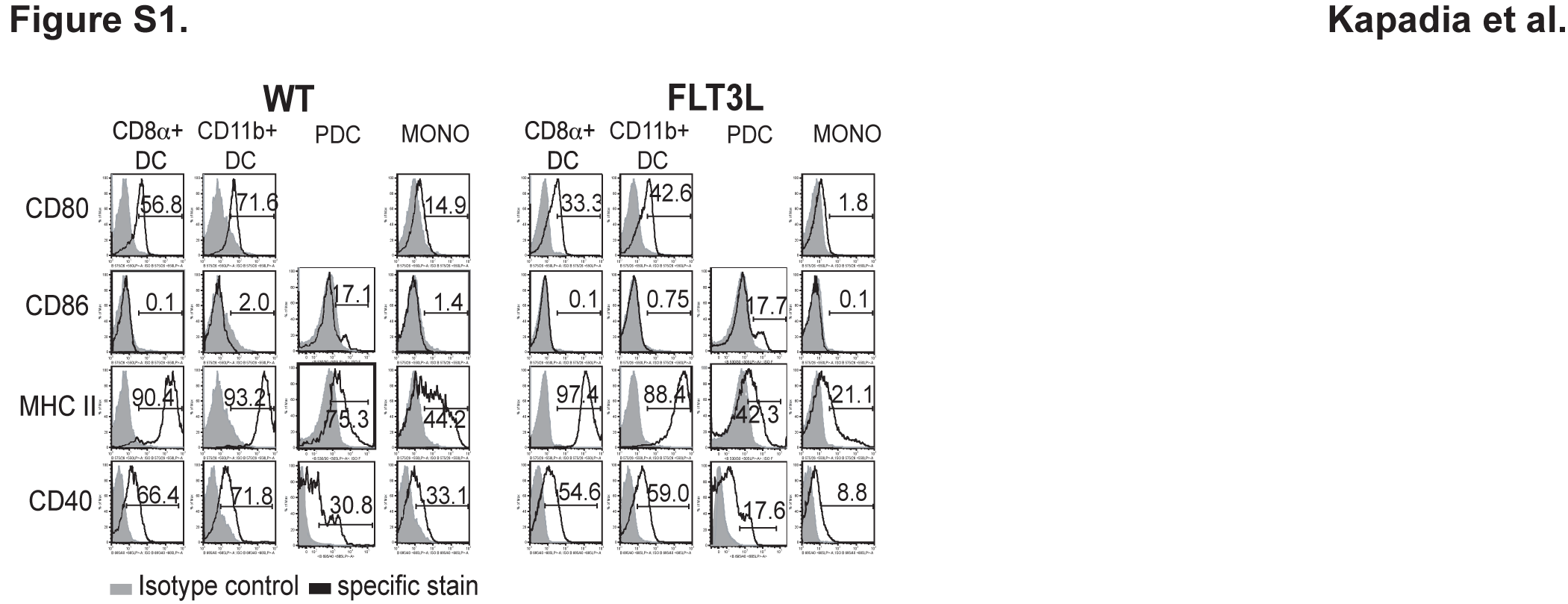

Supplement: Figure S1 — Phenotype of DCs from Flt3-L mobilized mice and DCs from unmobilized mice is similar. Total splenocytes isolated from Flt3-L mobilized mice and from wild-type mice were stained with antibodies against CD80, CD86, CD40 and MHC II. Cells were gated on DC subsets identified using the gating strategy described in Figure 1 A . Expression levels of these markers were determined using FlowJo software. Shaded histograms represent isotype control staining while empty histograms with solid line represent activation marker specific staining. Numbers over gates represent cell frequency. (TIF) [file pone.0019376.s001.tif]
